# ACAT1 and Metabolism-Related Pathways Are Essential for the Progression of Clear Cell Renal Cell Carcinoma (ccRCC), as Determined by Co-expression Network Analysis

**DOI:** 10.3389/fonc.2019.00957

**Published:** 2019-10-09

**Authors:** Liang Chen, Tianchen Peng, Yongwen Luo, Fenfang Zhou, Gang Wang, Kaiyu Qian, Yu Xiao, Xinghuan Wang

**Affiliations:** ^1^Department of Urology, Zhongnan Hospital of Wuhan University, Wuhan, China; ^2^Department of Biological Repositories, Zhongnan Hospital of Wuhan University, Wuhan, China; ^3^Laboratory of Precision Medicine, Zhongnan Hospital of Wuhan University, Wuhan, China

**Keywords:** clear cell renal cell carcinoma (ccRCC), weighted gene co-expression network analysis (WGCNA), survival prognosis, Fuhrman grade, ACAT1

## Abstract

Kidney cancer ranks as one of the top 10 causes of cancer death; this cancer is difficult to detect, difficult to treat, and poorly understood. The most common subtype of kidney cancer is clear cell renal cell carcinoma (ccRCC) and its progression is influenced by complex gene interactions. Few clinical studies have investigated the molecular markers associated with the progression of ccRCC. In this study, we collected microarray profiles of 72 ccRCCs and matched normal samples to identify differentially expressed genes (DEGs). Then a weighted gene co-expression network analysis (WGCNA) was conducted to identify co-expressed gene modules. By relating all co-expressed modules to clinical features, we found that the brown module and Fuhrman grade had the highest correlation (*r* = −0.8, *p* = 1e-09). Thus, the brown module was regarded as a clinically significant module and subsequently analyzed. Functional annotation showed that the brown module focused on metabolism-related biological processes and pathways, such as fatty acid oxidation and amino acid metabolism. We then performed a protein-protein interaction (PPI) network to identify the hub nodes in the brown module. It is worth noting that only one candidate, acetyl-CoA acetyltransferase (ACAT1), was considered to be the final target most relevant to the Fuhrman grade of ccRCC, by applying the intersection of hub genes in the co-expressed network and the PPI network. ACAT1 was subsequently validated using another two external microarray datasets and the TCGA dataset. Intriguingly, validation results indicated that ACAT1 was negatively correlated with four grades of ccRCC, which was also consistent with our results from qRT-PCR analysis and immunohistochemistry staining of clinical samples. Overexpression of ACAT1 inhibited the proliferation and migration of human ccRCC cells *in vitro*. In addition, the Kaplan-Meier survival curve showed that patients with a lower expression of ACAT1 showed a significantly lower overall survival rate and disease-free survival rate, indicating that ACAT1 could act as a prognostic and recurrence/progression biomarker of ccRCC. In summary, we found and confirmed that ACAT1 might help to identify the progression of ccRCC, which might have important clinical implications for enhancing risk stratification, therapeutic decision, and prognosis prediction in ccRCC patients.

## Introduction

Kidney cancer is one of the most common malignancies of the urinary system (1), ~90% of which is renal cell carcinoma (RCC). Clear cell RCC (ccRCC) accounts for between 70 and 85% of RCC, and has the highest rate of mortality (2). In the past decades, kidney cancer patients had few treatment options other than surgery, and 5-year survival was <20% once a metastatic disease developed (3, 4). A growing body of evidence shows a strong link between cancer and alerted metabolism. It is clear that many key oncogenic signaling pathways converge to accommodate tumor cell metabolism to support their growth and survival. In addition, some of these metabolic changes appear to be necessary for malignant transformation. In light of these basic findings, many researchers suggest that changes in cellular metabolism should be considered as an important marker of cancer (5). Previous studies have shown that seven known kidney cancer genes, VHL, MET, FLCN, TSC1, TSC2, FH, and SDH, are involved in pathways that respond to metabolic stress or nutrient stimulation, suggesting that kidney cancer is a disease of dysregulated cellular metabolism (6). There is, therefore, great significance in determining the effective metabolism-related biomarkers responsible for the genesis and development of ccRCC.

Currently, microarray and high-throughput sequencing technology have been widely applied to screen biomarkers of cancer (7, 8). The latest studies indicate that molecular biomarkers can improve the predictive accuracy of bladder cancer progression (9). Therefore, it is possible that we can identify such biomarkers that can predict the progression of renal cancer. Most of the studies focus on screening differentially expressed genes, but they have considerable limitations in ignoring the high correlations between genes. This finding may be functionally related between genes with similar expression patterns (10). The weighted gene co-expression network analysis (WGCNA) concentrates on the associations between genes, which is a systems biology method used to identify gene clusters associated with certain biological features (11). At present, similarly, this analysis is also used to identify tumor biomarkers, by correlating gene clusters with clinical features that can indicate tumor progression, such as tumor stage, grade, and metastasis (12, 13).

Thus, we intend to use the WGCNA method to find gene clusters related to ccRCC progression, which are indicated by clinical features, such as grade, stage, and metastasis. Some key metabolism-related genes and pathways can be identified from the gene clusters, which may illustrate the metabolic alteration during the progression of ccRCC (14, 15).

## Materials and Methods

### Data Preparation

Raw data were downloaded from public Gene Expression Omnibus (GEO, http://www.ncbi.nlm.nih.gov/geo/). Differentially expressed genes (DEGs) were identified from 72 ccRCC and 72 normal kidney samples using dataset GSE53757 performed on Affymetrix HG U133 Plus 2.0 (16). Thirty-nine samples from dataset GSE29609 performed on Agilent Whole Human Genome Oligo Microarray G4112A (Agilent-012391) were used to perform weighted gene co-expression networks. Two additional external data GSE40355 and GSE73731 (17) were used for validation. To obtain reliable results, we also applied the Oncomine database (http://www.oncomine.org/) and GEPIA (18) (Gene Expression Profiling Interactive Analysis) database based on TCGA (The Cancer Genome Atlas) for validation.

### Differentially Expressed Genes (DEGs)

Probes should be annotated first. We used “limma” (19) to screen the DEGs between ccRCC and normal samples. FDR (false discovery rate) <0.01 and |log_2_ (FC)| ≥1 were regarded as the cut-off criteria.

### Weighted Gene Co-expression Network

Detailed steps were described in our previous studies (20, 21). Briefly, the “WGCNA” (11) R package was used to conduct a co-expression network (GSE29609). To ensure the reliability of the constructed network, outlier samples were excluded after plotting a clustering dendrogram using the “flushClust” R package. After excluding outlier samples, a weighted adjacency matrix was generated by the formula amn = |cmn|^β^ (cmn represents Pearson's correlation between genes, amn represents adjacency between genes, the β parameter is based on the standard scale-free network and was used to magnify the correlation between genes). Then, a topological overlap matrix (TOM) was generated (22) and modules were identified by hierarchically clustering genes (23).

### Clinically Significant Modules and Module Functional Annotation

After the co-expressed network was generated, the correlations between modules and external clinical information were calculated using the Pearson correlation method. To further clarify the underlying mechanism of module genes in corresponding clinical features, genes of the interest module were enriched using the “clusterProfiler” (24) R package for functional and pathway enrichment analysis. A false discovery rate (FDR) <0.01 was considered to be statistically significant.

### Identifying Hub Genes

Based on the theory of WGCNA, hub genes had the highest degree of connectivity in a module, by which the biological significance of the module was determined. Connectivity and clinical trait relationship were measured by module connectivity and clinical trait relationship, as determined by the absolute value of the Pearson's correlation (cor.geneModuleMembership >0.8 and cor.geneTraitSignificance >0.2) (12). In addition, a protein-protein interaction (PPI) network of the clinically significant module was constructed using STRING (25) (Search Tool for the Retrieval of Interacting Genes, https://www.string-db.org/). The criterion for selecting hub nodes is a combined score of ≥0.8 and a connectivity degree of ≥20. To ensure the reliability of the identified hub genes, one possible strategy was to apply the intersection as the final target. Therefore, the “real” hub genes were taken as the intersection of the hub genes in the co-expression network and the hub nodes in the PPI network.

### Hub Gene Validation

To assess the correlation of hub gene expression in four distinct Fuhrman grades, we conducted a linear regression analysis using two additional independent validation datasets GSE40435 and GSE73731. In addition, RNA-seq data were obtained from the GEPIA (Gene Expression Profiling Interactive Analysis, http://gepia.cancer-pku.cn/) database to verify the association of hub gene expression with ccRCC progression. Survival plots for hub genes were also generated using the GEPIA database. Oncomine (https://www.oncomine.org) and the Human Protein Atlas database (26) (http://www.proteinatlas.org/) were used to verify mRNA and protein expression between tumor and normal samples.

### Cell Culture and Transfection

The human ccRCC cell lines ACHN and Caki1 were purchased from the Chinese Academy of Sciences in Shanghai. ACHN and Caki1 were cultured in Minimum Essential Medium (Gibco, China) and McCoy's 5A Medium (Gibco, China), respectively, both containing 10% fetal bovine serum (FBS) (Gibco, Australia) in a humidified atmosphere with 5% CO_2_ at 37°C. Human ccRCC cells were transfected with lentiviral vector (Control) and lentiviral ACAT1 (ACAT1) using Lipofectamine 3000 (Invitrogen, USA). The lentiviral vector (Catalog# EX-EGFP-Lv105) and lentiviral ACAT1 (Catalog# EX-C1085-Lv105) were purchased from GeneCopoeia, USA. Cells were transfected with 1 μg of the plasmid and incubated for 48 h before harvesting.

### Total RNA Isolation and qRT-PCR From Kidney Tissues

Total RNA from ccRCC and normal kidney tissues was isolated using a HiPure Total RNA Mini Kit (Magen, Shanghai, China), and quantified by NanoDrop. First-strand cDNA was synthesized using 1 μg of total RNA with a ReverTra Ace qPCR RT Kit (Toyobo, China). Each reaction was performed using 1 μg of the cDNAs with iQTM SYBR^®^ Green Supermix (Bio-Rad, Shanghai, China) in a final volume of 20 μl. ACAT1 primer: 5′-ATGCCAGTACACTGAATGATGG-3′ (forward), 5′-GATGCAGCATATACAGGAGCAA-3′ (reverse). GAPDH primer: 5′-TGCACCACCAACTGCTTAG-3′(forward), 5′-GATGCAGGGATGATGTTC-3′ (reverse). The annealing temperature of the two primers was 60°C.

### MTT Assay and Cloning Formation Assay

For the MTT assay, 3,000–5,000/200 μl medium transfected cells were seeded into 96-well dishes. Then, 20 μl of 5 mg/ml MTT reagent (Sigma-Aldrich) was added to each well, followed by incubation at 37°C for 4 h. After incubation, 100 μl of DMSO was added to each well to dissolve the precipitate. Absorbance at 570 nm was measured by a microplate reader (SpectraMax M2; Molecular Devices, USA). For the cloning formation assay, 1,000–1,500 transfected cells were seeded into six-well dishes to grow for 2 weeks. The cells were fixed with 4% PFA for 30 min, followed by staining with 0.1% crystal violet for 15 min.

### Transwell Assay

A 24-well plate Transwell chamber system was used to perform the Transwell assay. Next, 5–10 × 10^4^ transfected ccRCC cells in serum-free medium were seeded into the upper chamber (Corning, Inc. USA), while 10% FBS medium was added to the lower chamber. After incubation for 24 h at 37°C, the cells in the upper chamber were removed. Cells on the lower side of the chamber were fixed with 4% PFA for 30 min, followed by staining with 0.1% crystal violet, and the cells were photographed by microscopy.

### Western Blot Analysis

After transfection, cells were harvested and washed with cold PBS. Total protein was isolated using RIPA buffer with a protease inhibitor. A total of 20 μg of total protein was separated using 10% SDS-PAGE and transferred to PVDF membranes (Millipore, USA). After blocking with 5% bovine serum albumin (BSA), the membrane was incubated with primary antibodies overnight at 4°C. The membrane was then incubated with the second antibodies for 2 h at room temperature. We visualized the band using an enhanced chemiluminescence (ECL) kit (Bio-Rad) with a Bio-Rad ChemiDoc MP Imaging System (Bio-Rad, USA). GAPDH was used as a loading control. The primary antibodies were as follows: ant-ACAT1, 1:1,000 (Proteintech, catalog# 16215-1AP), anti-GAPDH, 1:1,000 (Santa Cruz, catalog# sc-365062).

### Immunofluorescence Staining and Evaluation for ccRCC Cells

Cells were seeded on coverslips after transfection. After washing with cold PBS, the cells were fixed with 4% PFA for 30 min and then treated with 0.1% Triton X-100 for 15 min. After blocking with 5% BSA for 30 min, the cells were incubated with Ki67 antibody (Novus, catalog# NBP2-19012) for 2 h at room temperature. After washing with PBS, the cells were incubated with Cy3-labeled secondary antibody for 1 h at room temperature. Immunofluorescence staining was visualized using a fluorescence microscope (Olympus, Japan) after nuclei were labeled with DAPI.

### Immunohistochemistry (IHC) Staining for ccRCC

The tissue microarray (TMA) of ccRCC was collaborated with Shanghai OutdoBiotech (Shanghai, China). The tissue microarray (catalog# HKidE180Su02) contained 150 ccRCC specimens and 30 adjacent normal tissues. The survival and clinical correlation analyses were based on the detailed clinical data of these 150 cases. Briefly, paraffin sections were hydrated and embedded, followed by incubation with 3% H_2_O_2_ for 15 min. Tissues were then incubated with citrate buffer for antigen retrieval. Tissues were incubated with ACAT1 antibody (Proteintech, catalog# 16215-1AP) after blocking with 5% BSA followed by incubation with biotinylated secondary antibody. Tissues were incubated with HRP substrate solution for 30 min, followed by incubation with DAB substrate chromogen solution. Tissue slides were counterstained with hematoxylin, dehydrated, and mounted. The ACAT1 staining signal was calculated based on the scores of the staining intensity and staining positive rate. The staining intensity is scored as follows: 0 points (negative), 1 point (weak), 2 points (moderate), and 3 points (strong). The staining positive rate is scored based on the positive cells as follows: 0 points (negative), 1 point (1–25%), 2 points (26–50%), 3 points (51–75%), and 4 points (76–100%). The ACAT1 total staining score is calculated by the formula: total score = staining intensity score × staining positive rate score. Total scores of ≥6 were regarded as a high expression and scores of <6 were regarded as a low expression.

### Gene Set Enrichment Analysis (GSEA)

To explore the functional role of ACAT1 in the progression of ccRCC, we performed gene set enrichment analysis (27) (GSEA, http://software.broadinstitute.org/gsea/index.jsp) using GSE73731. Based on the median expression value of ACAT1, 265 ccRCC tissue samples were divided into two groups. The reference sets were selected as c2.cp.kegg.v5.2.symbols.gmt to annotate all gene sets. In addition, the cut-off criteria for significantly enriched KEGG pathways were a gene size of ≥30, FDR of <0.05, and of an enrichment score (ES) |> 0.65.

## Results

### Differentially Expressed Genes in ccRCC Tissue Samples

**Figure 1:**
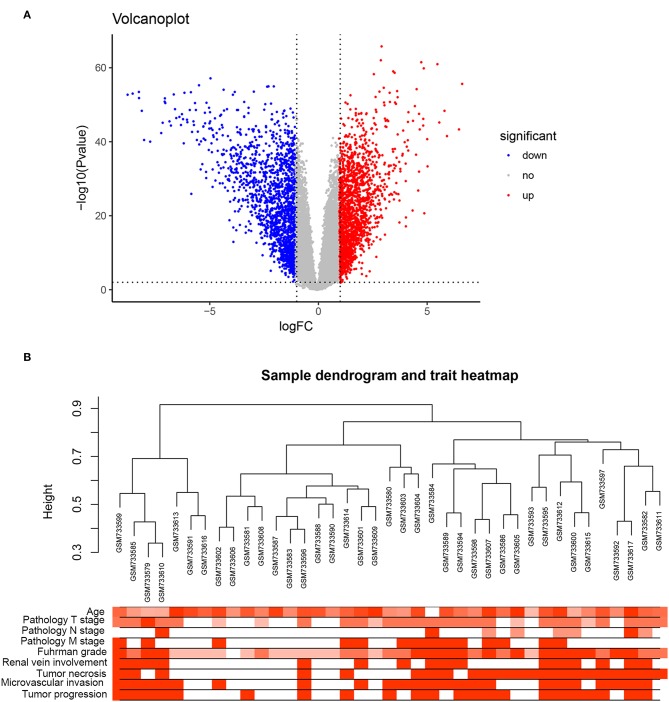
DEGs and clustering dendrogram of tumor samples, as well the clinical traits. **(A)** The volcano plot for all DEGs based on GSE53757. **(B)** The clustering was based on DEGs between ccRCC and normal. The red color represents positive renal vein involvement, tumor necrosis, microvascular invasion, and tumor progression. The color intensity represents older age, higher pathological stage, and Fuhrman grade.

Under the threshold of a false discovery rate (FDR) <0.01 and |log_2_ (FC)| ≥1, a total of 2,572 DEGs were identified between 72 ccRCC samples and 72 normal kidney samples. The volcano plot for all DEGs is shown in Figure 1A. All DEGs were selected for subsequent co-expression network construction. The flow chart of the study was shown in Figure S1.

### Weighted Co-expression Network

**Figure 2:**
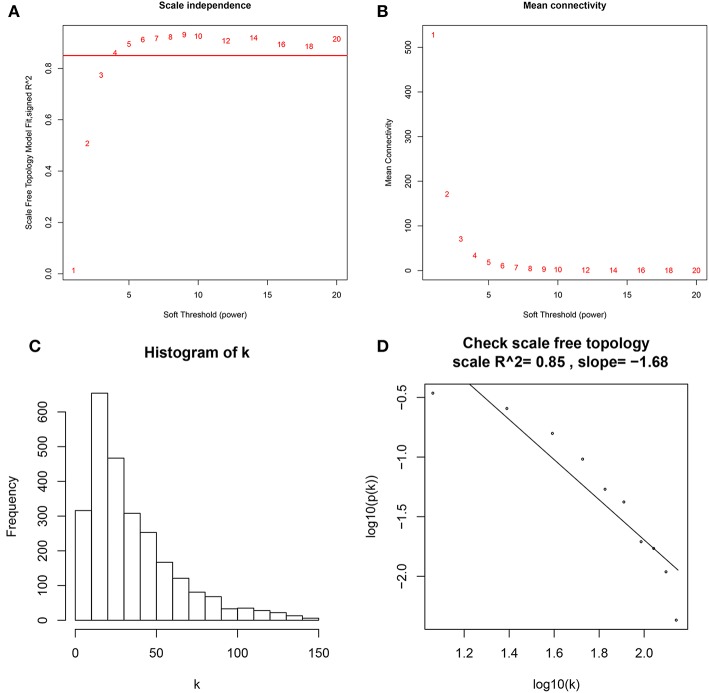
Determine soft-thresholding power in WGCNA. **(A)** The scale-free fit index for various soft-thresholding powers (β). **(B)** The mean connectivity for various soft-thresholding powers. **(C)** Histogram of connectivity distribution (β = 4). **(D)** Checking the scale free topology (β = 4).

**Figure 3:**
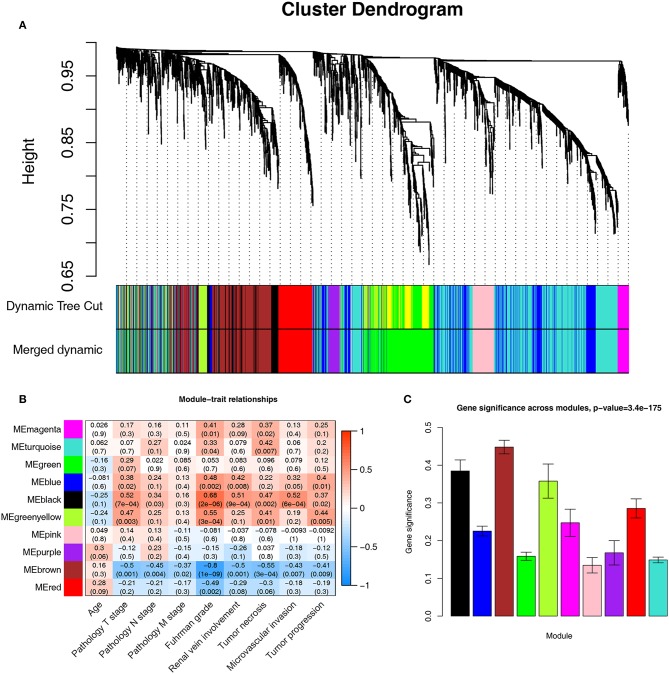
Identifying modules associated with the clinical traits of ccRCC. **(A)** Dendrogram of all DEGs clustered based on a dissimilarity measure (1-TOM). **(B)** Heatmap of the correlation between module eigengenes and clinical traits of ccRCC. **(C)** Distribution of average gene significance and errors in the modules associated with Fuhrman grade of ccRCC.

A co-expression network analysis was performed using 39 ccRCC samples in GSE29609 (Figure 1B). The 2,572 DEGs were included by adopting the “WGCNA” R package. β = 4 (scale free *R*^2^ = 0.85) was selected as the soft-thresholding power (Figures 2A–D). Ten modules were identified with a minimum size (gene group) of 30 for the gene dendrogram and a cut line of 0.25 for the module dendrogram (Figure 3A).

### Identification of Clinically Significant Module

Identifying the module most significantly associated with clinical features has considerable biological implications. The brown module has the highest correlation with the Fuhrman rank (*r* = −0.8, *p* = 1e-09, Figure 3B). In addition, the brown module also showed the highest gene significance associated with the Fuhrman grade (Figure 3C). Therefore, we chose the brown module as the module of interest and analyzed it.

**Figure 4:**
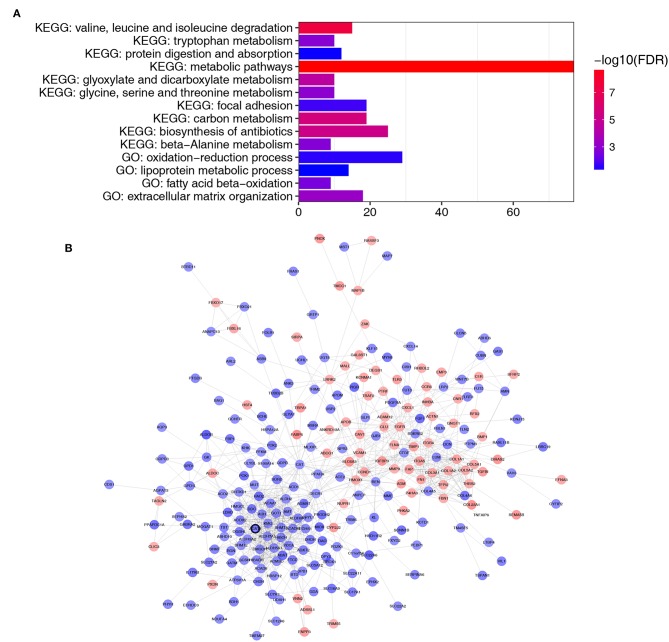
Functional enrichment and PPI network of genes in the brown module. **(A)** GO and pathway analysis of genes in the brown module. The x-axis represents the gene number and the represents the GO and KEGG terms. The color represents the –log10 (*P*-value) of each term. **(B)** PPI network of genes in the brown module. The red color represents upregulated genes and blue color represents downregulated genes. The bold circle labeled node represents common real hub gene in WGCNA and PPI network.

A total of 364 genes in the brown module were enriched for Gene Ontology (GO) and pathway analysis. Biological processes of the brown module were focused on fatty acid beta-oxidation, oxidation-reduction process and lipoprotein metabolic process (FDR <0.01). KEGG pathways of the brown module were significantly enriched in protein and carbon metabolism-related pathways (FDR <0.01, Figure 4A).

### Identification of Hub Genes

Thirty genes with high connectivity (cor.geneModuleMembership >0.8 and cor.geneTraitSignificance >0.2) in the brown module were selected as hub genes. Table 1 lists the hub genes that significantly correlated with Fuhrman grade. To identify the “real” hub genes, a protein-protein interaction (PPI) network was also constructed. The PPI network was visualized by Cytoscape (28) (Figure 4B). Genes with more than 20 nodes were regarded as hub nodes. Intriguingly, only one common hub gene, ACAT1, in both the co-expression network and PPI network was identified as a “real” hub gene and was further validated (Table 1).

**Table 1:** Hub genes in the module related to Fuhrman grade in co-expression and PPI network.

**Gene**	**Co-expression analysis**		**Hub gene in** **PPI network**	DEG analysis^*^	
	**cor.geneModule** **Membership**	**cor.geneTrai**t **Significance**		**logFC**	**FDR**
ACAT1	−0.861336	0.698151	Yes	−1.92496	1.97E-33
SLC6A13	−0.943927	0.773485	No	−1.72017	9.28E-10
DDAH1	−0.942308	0.744123	No	−1.15064	1.11E-25
FXYD2	−0.897976	0.736515	No	−1.2175	3.82E-15
EPHX2	−0.890283	0.702759	No	−2.10646	1.58E-28
BBOX1	−0.890081	0.786987	No	−1.76985	1.88E-14
LRP2	−0.888462	0.693115	No	−1.07557	8.58E-08
GBA3	−0.88583	0.795882	No	−1.71742	2.19E-11
DDC	−0.880567	0.747123	No	−3.296	1.02E-26
SLC5A10	−0.871862	0.766412	No	−1.62937	2.60E-10
PDZK1	−0.869838	0.791488	No	−1.41303	2.28E-15
ABHD6	−0.865587	0.728156	No	−1.02397	1.09E-17
AGXT2	−0.861336	0.656894	No	−2.64568	3.19E-16
GATM	−0.856275	0.69933	No	−2.41321	2.40E-29
C11orf54	−0.848988	0.687114	No	−1.60588	6.19E-26
ANK3	−0.848583	0.659466	No	−1.94172	1.07E-21
CXCL14	−0.840486	0.56838	No	−1.12772	1.54E-11
LGALS2	−0.838866	0.687221	No	−1.2955	1.70E-08
APOM	−0.837652	0.692364	No	−2.88154	7.87E-27
RBP5	−0.831984	0.616709	No	−1.48516	1.73E-12
CLDN10	−0.831781	0.770056	No	−2.39593	2.49E-34
MAP7	−0.827935	0.722477	No	−1.3653	5.00E-16
FBXL16	−0.824696	0.679612	No	1.822383	1.20E−15
DEPDC7	−0.815992	0.698687	No	−1.17135	1.76E-09
CRYL1	−0.814372	0.632783	No	−1.7183	5.71E-32
EMX2OS	−0.811741	0.715404	No	−1.06888	6.73E-11
LEPROTL1	−0.810324	0.727192	No	1.014621	1.07E-26
UPB1	−0.809514	0.589276	No	−1.26555	7.37E-07
CLCN5	−0.80668	0.534731	No	−2.05668	1.03E-30
WDR72	−0.803036	0.605886	No	−2.64714	1.78E-23

* *Differentially expressed genes between 72 ccRCC tissues and 72 normal kidney tissues in GSE53757*.

### Hub Gene Validation

**Figure 5:**
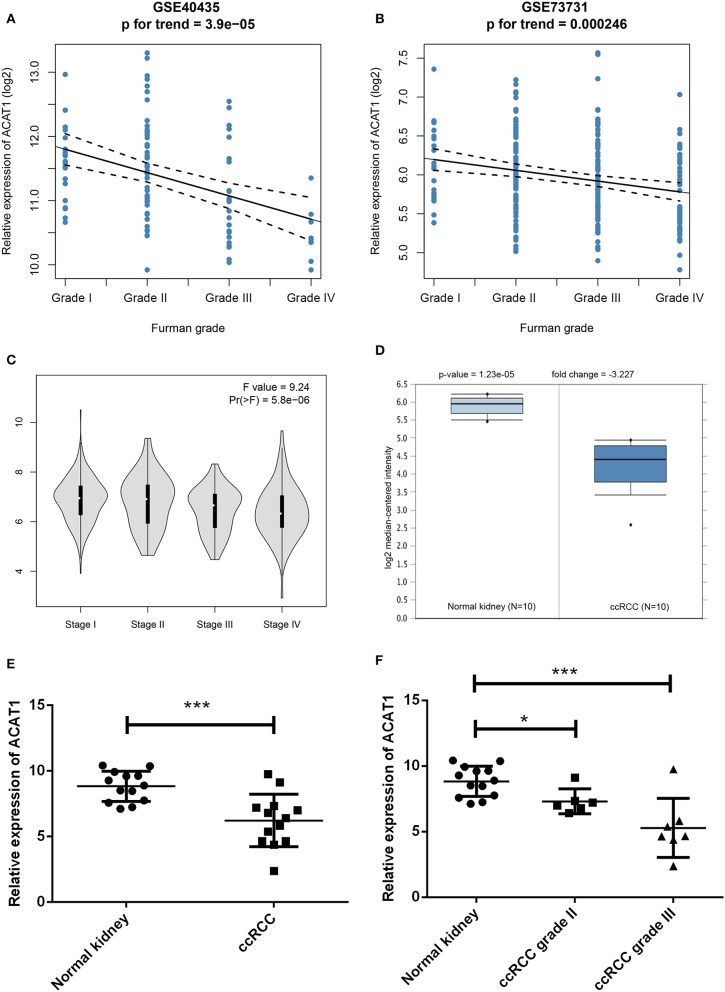
Validation of hub gene. **(A)** The correlation of ACAT1 expression with the Fuhrman grade of ccRCC (based on microarray data of GSE40435). **(B)** The correlation of ACAT1 expression with the Fuhrman grade of ccRCC (based on microarray data of GSE73731). **(C)** The violin plot of ACAT1 across different pathological stages based on the TCGA data in the GEPIA database. **(D)** ACAT1 mRNA expression in ccRCC tissue samples (*n* = 10) and normal kidney tissues (*n* = 10) based on the Oncomine database. **(E)** qRT-PCR results suggested that ACAT1 mRNA expression was low in 13 ccRCC tissue samples compared with 13 matched normal kidneys. **(F)** ACAT1 mRNA expression in different grades of ccRCC and matched normal kidney samples indicated by qRT-PCR. ^*^p < 0.05, ^**^p < 0.01, ^***^p < 0.001.

**Figure 6:**
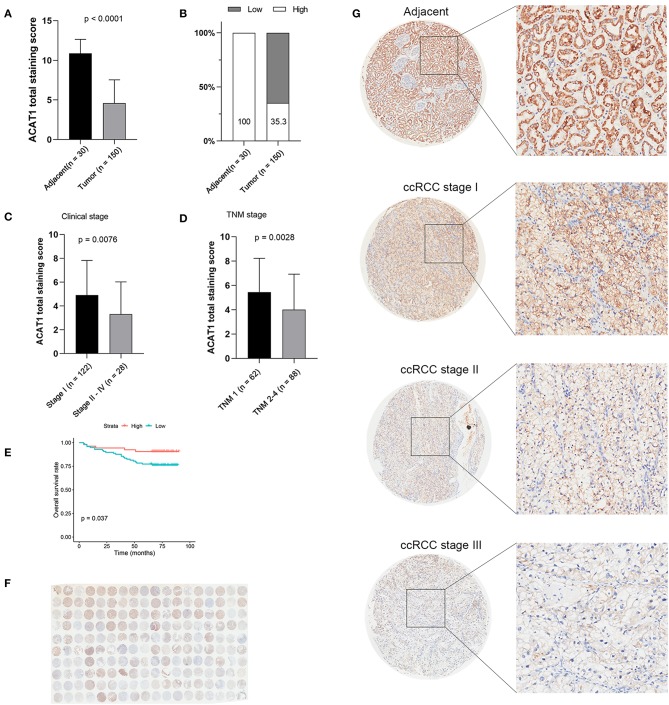
Tissue microarray staining results confirmed that ACAT1 protein was downregulated in ccRCC tissues. **(A)** Total staining scores of ACAT1 in 150 ccRCC tissues and 30 adjacent normal tissues. **(B)** Percentage of low and high ACAT1 staining scores in ccRCC and adjacent tissues. **(C)** Total staining score of ACTA1 in ccRCC with clinical stage I and stage II–IV. **(D)** Total staining score of ACTA1 in ccRCC with TNM 1 and TNM 2–4. **(E)** Kaplan-Meier plot of overall survival for ACAT1 expression based on the tissue microarray data. **(F)** ACAT1 staining of ccRCC tissue microarray. **(G)** Representative pattern of ACAT1 protein expression in adjacent normal tissues and ccRCC tissues using tissue microarray sections.

Another three independent datasets were used to verify the expression of ACAT1 in different Fuhrman grades. In test set GSE40435 and GSE73731, linear regression analyses showed that ACAT1 expression had a strongly negative correlation with a Fuhrman grade of ccRCC (Figures 5A,B). The results from the clinical samples also suggested that ACAT1 mRNA and protein were both decreased in ccRCC (Figures 5D–F, 6A,B, S2A–D). In addition, the results from the qPCR and tissue microarray of ccRCC showed that ACAT1 expression at both the mRNA and protein level is lower in high stage ccRCC than in low stage ccRCC (Figures 5C,F, 6C,D,G, Table 2). Furthermore, ccRCC patients with lower ACAT1 expression had significantly shorter overall survival (OS) and disease-free survival (DFS) times (Figure 6E and Figures S2E,F).

**Table 2:** Association between ACAT1 expression and clinicopathological features of human ccRCC.

**variables**	**ACAT1 expression in human ccRCC tissues**				
	**Cases (*n* = 150)**	**High**	**Low**	**χ^2^**	***P*-value**
**Age**					
≤65 y	120	46	74	2.36	0.12
>65 y	30	7	23		
**Gender**					
Female	43	17	26	0.47	0.49
Male	107	36	71		
**Tumor size(cm)**					
<7 cm	119	48	71	6.31	0.012^*^
≥7 cm	31	5	26		
**TNM stage**					
TNM1	62	27	35	3.12	0.077
TNM2–4	88	26	62		
**Clinical stage^#^**					
Stage I	122	47	75	2.91	0.087
Stage II–IV	28	6	22		
**Survival status**					
Live	122	48	74	4.60	0.032^*^
Dead	28	5	23		

# *Based on AJCC 7th edition*.

* *p < 0.05 is considered significant*.

### Overexpression of ACAT1 Inhibited Proliferation and Migration of ccRCC Cells

**Figure 7:**
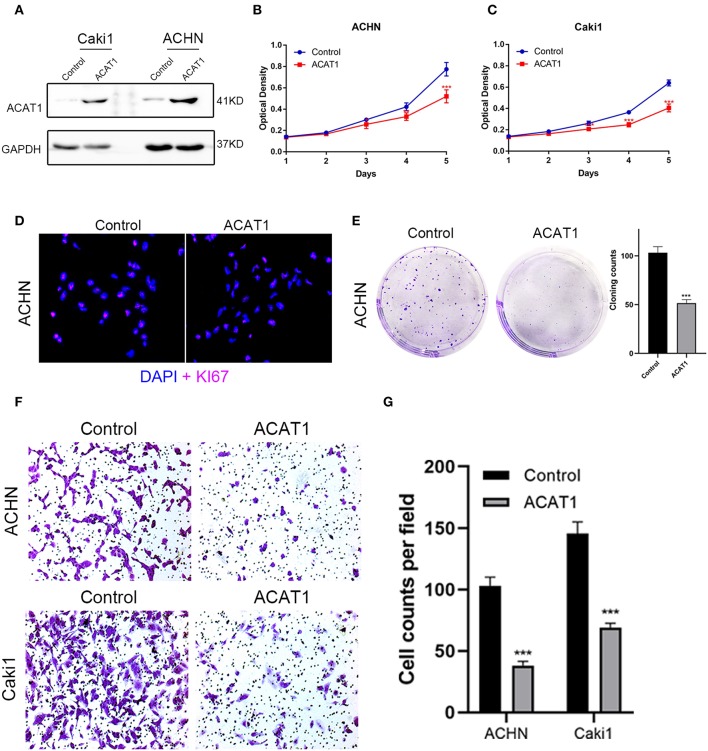
Overexpressed ACAT1 inhibited proliftion and migration of ccRCC cells. **(A)** Overexpressed ACAT1 increased ACAT1 protein by western blot analysis. GAPDH abundance was used as an internal control. **(B–C)** Distinct ccRCC cells transfected with empty control or lentiviral ACAT1 were allowed to grow at the indicated times, and the cell viability was determined by the MTT assay. **(D)** Representative Ki-67 staining (red) revealed the cell proliferation of ccRCC cells after ACAT1 overexpression or empty control treatment. Nuclei were counterstained by DAPI (blue). **(E)** The cell survival was measured by the cloning formation assay after transfected with empty control or lentiviral ACAT1. **(F)** The cell migration was measured by Transwell assay after transfected with empty control or lentiviral ACAT1. **(G)** The statistical results of the Transwell migration assay. ^*^p < 0.05, ^**^p < 0.01, ^***^p < 0.001.

To investigate the effect of ACAT1 on the viability and proliferation of ccRCC cells, ACHN and Caki1 cells were treated with lentiviral control and lentiviral ACAT1 for 48 h and determined by MTT assay and cloning formation assay, suggesting that overexpression of ACAT1 drastically restrained ccRCC cell proliferation (Figures 7A–E). Immunofluorescence staining revealed that the ACAT1-overexpression group showed fewer Ki-67-positive cells than the control group (Figure 7D). Transwell migration assays showed that overexpression of ACAT1 reduced the cell migration of ccRCC cells (Figures 7F,G).

### Functional Annotation for the Hub Gene

**Figure 8:**
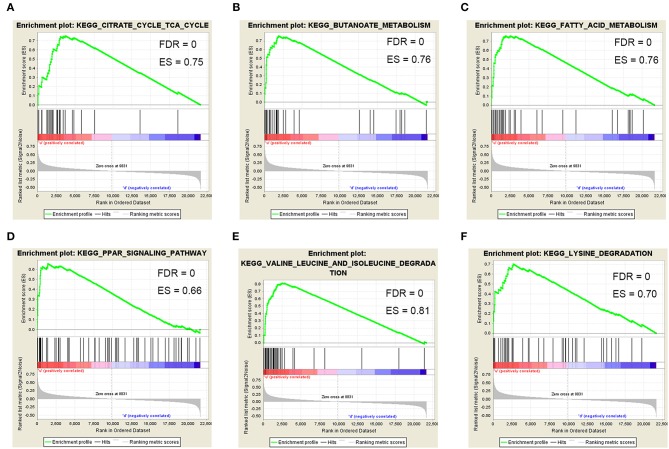
Gene set enrichment analysis (GSEA). Six representative functional gene sets enriched in ccRCC with ACAT1 downregulated based on GSE73731. **(A)** Citrate cycle TCA cycle. **(B)** Butanoate metabolism. **(C)** Fatty acid metabolism. **(D)** PPAR signaling pathway. **(E)** Valine leucine and isoleucine degradation. **(F)** Lysine degradation.

To determine the functional role of ACAT1 in ccRCC progression, we performed a GSEA analysis using 265 ccRCC samples. Under the cut-off criteria of gene size ≥30, FDR <0.05, and |enrichment score (ES) |>0.75, six pathways were significantly enriched. Interestingly, the pathways were all involved in lipid and fatty acid metabolism processes, including “fatty acid metabolism,” “valine leucine and isoleucine degradation,” “PPAR signaling pathway,” “lysine degradation,” “butanoate metabolism,” and “citrate cycle TCA cycle” (Figure 8).

## Discussion

ccRCC is the most common subtype of kidney cancer and its progression is affected by complex gene interactions (29). Many studies suggest that altered cellular metabolism is an important marker of cancer (5). Previous studies have shown that kidney cancer is a disease of dysregulated cellular metabolism (5). Determining effective metabolism-related biomarkers responsible for the genesis and development of ccRCC is strongly warranted. Molecular biomarkers associated with ccRCC progression have been predicted in many studies. By comparing ccRCC at different histological levels, eight genes were identified to distinguish between different levels of ccRCC (30). This analytical method might, however, result in large false positive results because it did not use a global level system biological analysis method. By directly comparing gene expression, other studies found that EphA1 (31), EphA2 (32), and VEGFR-1 (33) were indicators in different stages of ccRCC but lacked large sample support.

Unlike these researches, we used a systems biology method combined with a large number of samples to screen specific biomarkers of ccRCC. Most importantly, molecular biomarkers should be well-differentiated between tumor and normal tissues. On this basis, the co-expression network was performed by the dynamic tree cutting method, and 10 co-expression modules were identified. Correlation analysis showed that the brown module had the highest correlation with the Fuhrman grade among the 10 modules. Hub genes with the highest connectivity were identified from the brown module, which determined the characteristics of the module to a large extent. In addition, a PPI network was constructed based on the genes in the brown module, and hub nodes were also identified. Finally, only one common hub gene, ACAT1, was selected as the real hub gene in the co-expression network and PPI network for further validation. Further verification also confirmed that ACAT1 was negatively correlated with the Fuhrman grade of ccRCCs, and its expression was also related to overall survival and the disease-free survival of ccRCC patients.

The ACAT1 enzyme carries out the last step in ketone breakdown during fasting or starving. The enzyme could catalyze the reversible formation of acetoacetyl-CoA from two molecules of acetyl-CoA (34). Ketone bodies are an important source of energy during fasting in normal cells. ACAT1 gene mutation induced a deficiency in mitochondrial acetoacetyl-CoA thiolase, which was also called ketothiolase deficiency (35). The most important fuel that supports tumor growth is carbohydrates. It therefore seems reasonable that a low-carbohydrate diet could reduce the progression of cancer. Many mouse studies have shown that dietary restriction reduced tumor size and growth rate (36), prolonged survival (37), and increased sensitivity to radiotherapy (36). Therefore, we can assume that the decreased ACAT1 expression in high-grade ccRCC may be caused by metabolic changes, as the invasive tumors cannot obtain enough energy from ketolysis and fatty acid oxidation to support their growth.

Compared with benign prostate tissues, ACAT1 expression was significantly increased in prostate cancer tissues (38, 39). Upregulation of ACAT1 was correlated with more aggressive pancreatic cancer. Interestingly, Zhao et al. (40) and White et al. (41) both performed quantitative proteomic analysis and identified that the ACAT1 protein was decreased in ccRCC compared with adjacent tissues, which was consistent with our findings.

Previous studies reported that altered pathways, such as metabolic pathways (42), glycolysis, and fatty acid oxidation (43), were confirmed by our results from the brown module, revealing that metabolism changes were important for ccRCC progression. Lipid droplets were often found in ccRCC cytoplasm (4); therefore, ACAT1 may participate in cholesterol metabolism, similar to cytosolic acetyl-CoA acetyltransferase 2 (ACAT2), which indicated that lipid and fatty acid metabolism were different between kidney cancer and prostate cancer, as well as pancreatic cancer. In our study, ACAT1 at the transcriptional and translational levels were significantly decreased in ccRCC tissues (Figures 5D, 6). Moreover, ACAT1 had a strong negative correlation with the four grades of ccRCC, indicating that ACAT1 was closely associated with the progression of ccRCC. Notably, when ccRCC patients had lower expressions of ACAT1, they exhibited a significantly shorter OS and DFS rate. Furthermore, an *in vitro* study indicated that overexpressed ACAT1 inhibited the proliferation and migration of renal cell ACHN and Caki1 cells, suggesting that ACAT1 might be a favorable prognostic marker in ccRCC (Figures 5E,F).

We should also consider some of the limitations of this study. Our findings should be validated using a larger number of clinical samples. In addition, the mechanisms governing the impact of ACAT1 on the progression of ccRCC should be elucidated by molecular biology experiments, which is our next research plan. In conclusion, we built a co-expression network and identified the ACAT1 related to the progression of ccRCC, which might have important clinical significance in improving risk stratification, treatment decision-making and prognosis prediction in ccRCC patients.

## Data Availability Statement

Publicly available datasets were analyzed in this study. This data can be found here: https://www.ncbi.nlm.nih.gov/geo/query/acc.cgi?acc=GSE53757, https://www.ncbi.nlm.nih.gov/geo/query/acc.cgi?acc=GSE29609, https://www.ncbi.nlm.nih.gov/geo/query/acc.cgi?acc=GSE40435, https://www.ncbi.nlm.nih.gov/geo/query/acc.cgi?acc=GSE73731.

## Ethics Statement

Written informed consent was obtained from all participants of this study. All participants were over 16 years old. The study using human kidney tissue samples for RNA isolation was approved by the Ethics Committee of Zhongnan Hospital of Wuhan University (approval number: 2015029) (44).

## Author Contributions

LC, TP, YL, YX, and XW conceived and designed the study. LC, TP, YL, and YX performed the analysis procedures. LC, FZ, GW, and YX analyzed the results. YL, KQ, and YX contributed analysis tools. LC and TP performed the experiments. LC, TP, YL, and YX contributed to the writing of the manuscript. All authors reviewed the manuscript.

### Conflict of Interest

The authors declare that the research was conducted in the absence of any commercial or financial relationships that could be construed as a potential conflict of interest.

## Supplementary Material

Figure S1 Flow chart of data collection, preparation, processing, analysis, and validation in this study.

Figure S2 ACAT1 decreased at both the transcriptional and translational levels in ccRCC. **(A,C)** Immunohistochemistry staining of ACAT1 in normal kidney tissues. **(B,D)** Immunohistochemistry staining of ACAT1 in ccRCC tissues. **(E,F)** Kaplan-Meier plot of overall survival time **(E)** and disease-free survival time **(F)** for ACAT1 based on TCGA data in the GEPIA database.

## Abbreviations

WGCNA: Weighted Gene Co-expression Network Analysis
ccRCC: Clear Cell Renal Cell Carcinoma
FC: Fold Change
GO: Gene Ontology
PPI: Protein-Protein Interaction
BP: Biological Process
GSEA: Gene Set Enrichment Analysis
FDR: False Discovery Rate
FC: Fold Change
TOM: Topological Overlap Matrix
OS: Overall Survival
DFS: Disease-free Survival.

## References

[1] Siegel RL Miller KD Jemal A Cancer Statistics, 2017. CA Cancer J Clin. (2017) 67:7–30. 10.3322/caac.21387 28055103

[2] Cairns P Renal cell carcinoma. Cancer Biomark. (2010) 9:461–73. 10.3233/CBM-2011-0176 22112490 PMC3308682

[3] Mekhail TM Abou-Jawde RM Boumerhi G Malhi S Wood L Elson P. Validation and extension of the Memorial Sloan-Kettering prognostic factors model for survival in patients with previously untreated metastatic renal cell carcinoma. J Clin Oncol. (2005) 23:832–41. 10.1200/JCO.2005.05.179 15681528

[4] Rini BI Campbell SC Escudier B Renal cell carcinoma. Lancet. (2009) 373:1119–32. 10.1016/S0140-6736(09)60229-4 19269025

[5] Cairns RA Harris IS Mak TW. Regulation of cancer cell metabolism. Nat Rev Cancer. (2011) 11:85–95. 10.1038/nrc2981 21258394

[6] Linehan WM Srinivasan R Schmidt LS. The genetic basis of kidney cancer: a metabolic disease. Nat Rev Urol. (2010) 7:277–85. 10.1038/nrurol.2010.47 20448661 PMC2929006

[7] Dahinden C Ingold B Wild P Boysen G Luu VD Montani M. Mining tissue microarray data to uncover combinations of biomarker expression patterns that improve intermediate staging and grading of clear cell renal cell cancer. Clin Cancer Res. (2010) 16:88–98. 10.1158/1078-0432.CCR-09-0260 20028743

[8] Gerlinger M Horswell S Larkin J Rowan AJ Salm MP Varela I. Genomic architecture and evolution of clear cell renal cell carcinomas defined by multiregion sequencing. Nat Genet. (2014) 46:225–33. 10.1038/ng.2891 24487277 PMC4636053

[9] van Kessel KEM van der Keur KA Dyrskjot L Algaba F Welvaart NYC Beukers W. Molecular markers increase precision of the european association of urology non-muscle-invasive bladder cancer progression risk groups. Clin Cancer Res. (2018) 24:1586–93. 10.1158/1078-0432.CCR-17-2719 29367430

[10] Tavazoie S Hughes JD Campbell MJ Cho RJ Church GM. Systematic determination of genetic network architecture. Nat Genet. (1999) 22:281–5. 10.1038/10343 10391217

[11] Langfelder P Horvath S. WGCNA: an R package for weighted correlation network analysis. BMC Bioinformatics. (2008) 9:559. 10.1186/1471-2105-9-559 19114008 PMC2631488

[12] Chen L Yuan L Wang Y Wang G Zhu Y Cao R. Co-expression network analysis identified FCER1G in association with progression and prognosis in human clear cell renal cell carcinoma. Int J Biol Sci. (2017) 13:1361–72. 10.7150/ijbs.21657 29209141 PMC5715520

[13] Chen P Wang F Feng J Zhou R Chang Y Liu J. Co-expression network analysis identified six hub genes in association with metastasis risk and prognosis in hepatocellular carcinoma. Oncotarget. (2017) 8:48948–58. 10.18632/oncotarget.16896 28430663 PMC5564739

[14] Clarke C Madden SF Doolan P Aherne ST Joyce H O'Driscoll L. Correlating transcriptional networks to breast cancer survival: a large-scale coexpression analysis. Carcinogenesis. (2013) 34:2300–8. 10.1093/carcin/bgt208 23740839

[15] Chou WC Cheng AL Brotto M Chuang CY. Visual gene-network analysis reveals the cancer gene co-expression in human endometrial cancer. BMC Genomics. (2014) 15:300. 10.1186/1471-2164-15-300 24758163 PMC4234489

[16] von Roemeling CA Radisky DC Marlow LA Cooper SJ Grebe SK Anastasiadis PZ. Neuronal pentraxin 2 supports clear cell renal cell carcinoma by activating the AMPA-selective glutamate receptor-4. Cancer Res. (2014) 74:4796–810. 10.1158/0008-5472.CAN-14-0210 24962026 PMC4154999

[17] Wei X Choudhury Y Lim WK Anema J Kahnoski RJ Lane B. Recognizing the continuous nature of expression heterogeneity and clinical outcomes in clear cell renal cell carcinoma. Sci Rep. (2017) 7:7342. 10.1038/s41598-017-07191-y 28779136 PMC5544702

[18] Tang Z Li C Kang B Gao G Li C Zhang Z. GEPIA: a web server for cancer and normal gene expression profiling and interactive analyses. Nucleic Acids Res. (2017) 45:W98–102. 10.1093/nar/gkx247 28407145 PMC5570223

[19] Ritchie ME Phipson B Wu D Hu Y Law CW Shi W. limma powers differential expression analyses for RNA-sequencing and microarray studies. Nucleic Acids Res. (2015) 43:e47. 10.1093/nar/gkv007 25605792 PMC4402510

[20] Chen L Yuan L Qian K Qian G Zhu Y Wu CL. Identification of biomarkers associated with pathological stage and prognosis of clear cell renal cell carcinoma by co-expression network analysis. Front Physiol. (2018) 9:399. 10.3389/fphys.2018.00399 29720944 PMC5915556

[21] Yuan L Qian G Chen L Wu CL Dan HC Xiao Y. Co-expression network analysis of biomarkers for adrenocortical carcinoma. Front Genet. (2018) 9:328. 10.3389/fgene.2018.00328 30158955 PMC6104177

[22] Yip AM Horvath S. Gene network interconnectedness and the generalized topological overlap measure. BMC Bioinformatics. (2007) 8:22. 10.1186/1471-2105-8-22 17250769 PMC1797055

[23] Ravasz E Somera AL Mongru DA Oltvai ZN Barabasi AL. Hierarchical organization of modularity in metabolic networks. Science. (2002) 297:1551–5. 10.1126/science.1073374 12202830

[24] Yu G Wang LG Han Y He QY. clusterProfiler: an R package for comparing biological themes among gene clusters. OMICS. (2012) 16:284–7. 10.1089/omi.2011.0118 22455463 PMC3339379

[25] Szklarczyk D Franceschini A Wyder S Forslund K Heller D Huerta-Cepas J. STRING v10: protein-protein interaction networks, integrated over the tree of life. Nucleic Acids Res. (2015) 43:D447–52. 10.1093/nar/gku1003 25352553 PMC4383874

[26] Uhlen M Fagerberg L Hallstrom BM Lindskog C Oksvold P Mardinoglu A. Proteomics. Tissue-based map of the human proteome. Science. (2015) 347:1260419. 10.1126/science.1260419 25613900

[27] Subramanian A Tamayo P Mootha VK Mukherjee S Ebert BL Gillette MA. Gene set enrichment analysis: a knowledge-based approach for interpreting genome-wide expression profiles. Proc Natl Acad Sci USA. (2005) 102:15545–50. 10.1073/pnas.0506580102 16199517 PMC1239896

[28] Shannon P Markiel A Ozier O Baliga NS Wang JT Ramage D. Cytoscape: a software environment for integrated models of biomolecular interaction networks. Genome Res. (2003) 13:2498–504. 10.1101/gr.1239303 14597658 PMC403769

[29] Creighton CJ Morgan M Gunaratne PH Wheeler DA Gibbs RA Robertson A Comprehensive molecular characterization of clear cell renal cell carcinoma. Nature. (2013) 499:43–9. 10.1038/nature12222 23792563 PMC3771322

[30] Wan F Zhu Y Han C Xu Q Wu J Dai B. Identification and validation of an eight-gene expression signature for predicting high Fuhrman grade renal cell carcinoma. Int J Cancer. (2017) 140:1199–208. 10.1002/ijc.30535 27874173

[31] Wang X Liu Y Cao G Zhang X Xu H Xu H. Expression of the EphA1 protein is associated with Fuhrman nuclear grade in clear cell renal cell carcinomas. Int J Clin Exp Pathol. (2015) 8:6821–7. 26261568 PMC4525902

[32] Wang L Hu H Tian F Zhou W Zhou S Wang J. Expression of EphA2 protein is positively associated with age, tumor size and Fuhrman nuclear grade in clear cell renal cell carcinomas. Int J Clin Exp Pathol. (2015) 8:13374–80. 26722543 PMC4680488

[33] Lkhagvadorj S Oh SS Lee MR Jung JH Chung HC Cha SK. VEGFR-1 expression relates to Fuhrman nuclear grade of clear cell renal cell carcinoma. J Lifestyle Med. (2014) 4:64–70. 10.15280/jlm.2014.4.1.64 26064856 PMC4390762

[34] Kano M Fukao T Yamaguchi S Orii T Osumi T Hashimoto T. Structure and expression of the human mitochondrial acetoacetyl-CoA thiolase-encoding gene. Gene. (1991) 109:285–90. 10.1016/0378-1119(91)90623-J 1684944

[35] Francis T Wartofsky L. Common thyroid disorders in the elderly. Postgrad Med. (1992) 92:225–30, 233–6. 10.1080/00325481.1992.11701452 1518756

[36] Morscher RJ Aminzadeh-Gohari S Feichtinger RG Mayr JA Lang R Neureiter D. Inhibition of neuroblastoma tumor growth by ketogenic diet and/or calorie restriction in a CD1-Nu mouse model. PLoS ONE. (2015) 10:e0129802. 10.1371/journal.pone.0129802 26053068 PMC4459995

[37] Poff AM Ari C Seyfried TN D'Agostino DP. The ketogenic diet and hyperbaric oxygen therapy prolong survival in mice with systemic metastatic cancer. PLoS ONE. (2013) 8:e65522. 10.1371/journal.pone.0065522 23755243 PMC3673985

[38] Saraon P Cretu D Musrap N Karagiannis GS Batruch I Drabovich AP. Quantitative proteomics reveals that enzymes of the ketogenic pathway are associated with prostate cancer progression. Mol Cell Proteomics. (2013) 12:1589–601. 10.1074/mcp.M112.023887 23443136 PMC3675816

[39] Saraon P Trudel D Kron K Dmitromanolakis A Trachtenberg J Bapat B. Evaluation and prognostic significance of ACAT1 as a marker of prostate cancer progression. Prostate. (2014) 74:372–80. 10.1002/pros.22758 24311408

[40] Zhao Z Wu F Ding S Sun L Liu Z Ding K. Label-free quantitative proteomic analysis reveals potential biomarkers and pathways in renal cell carcinoma. Tumour Biol. (2015) 36:939–51. 10.1007/s13277-014-2694-2 25315187

[41] White NM Masui O Desouza LV Krakovska O Metias S Romaschin AD. Quantitative proteomic analysis reveals potential diagnostic markers and pathways involved in pathogenesis of renal cell carcinoma. Oncotarget. (2014) 5:506–18. 10.18632/oncotarget.1529 24504108 PMC3964225

[42] Perroud B Lee J Valkova N Dhirapong A Lin PY Fiehn O. Pathway analysis of kidney cancer using proteomics and metabolic profiling. Mol Cancer. (2006) 5:64. 10.1186/1476-4598-5-64 17123452 PMC1665458

[43] Atrih A Mudaliar MA Zakikhani P Lamont DJ Huang JT Bray SE. Quantitative proteomics in resected renal cancer tissue for biomarker discovery and profiling. Br J Cancer. (2014) 110:1622–33. 10.1038/bjc.2014.24 24548857 PMC3960606

[44] Cao R Meng Z Liu T Wang G Qian G Cao T. Decreased TRPM7 inhibits activities and induces apoptosis of bladder cancer cells via ERK1/2 pathway. Oncotarget. (2016) 7:72941–60. 10.18632/oncotarget.12146 27662662 PMC5341955

