# Supplementary figures and images for: ACAT1 and Metabolism-Related Pathways Are Essential for the Progression of Clear Cell Renal Cell Carcinoma (ccRCC), as Determined by Co-expression Network Analysis
Source: Front Oncol. 2019 Oct 9;9:957. doi: 10.3389/fonc.2019.00957 (PMC6795108; doi:10.3389/fonc.2019.00957)

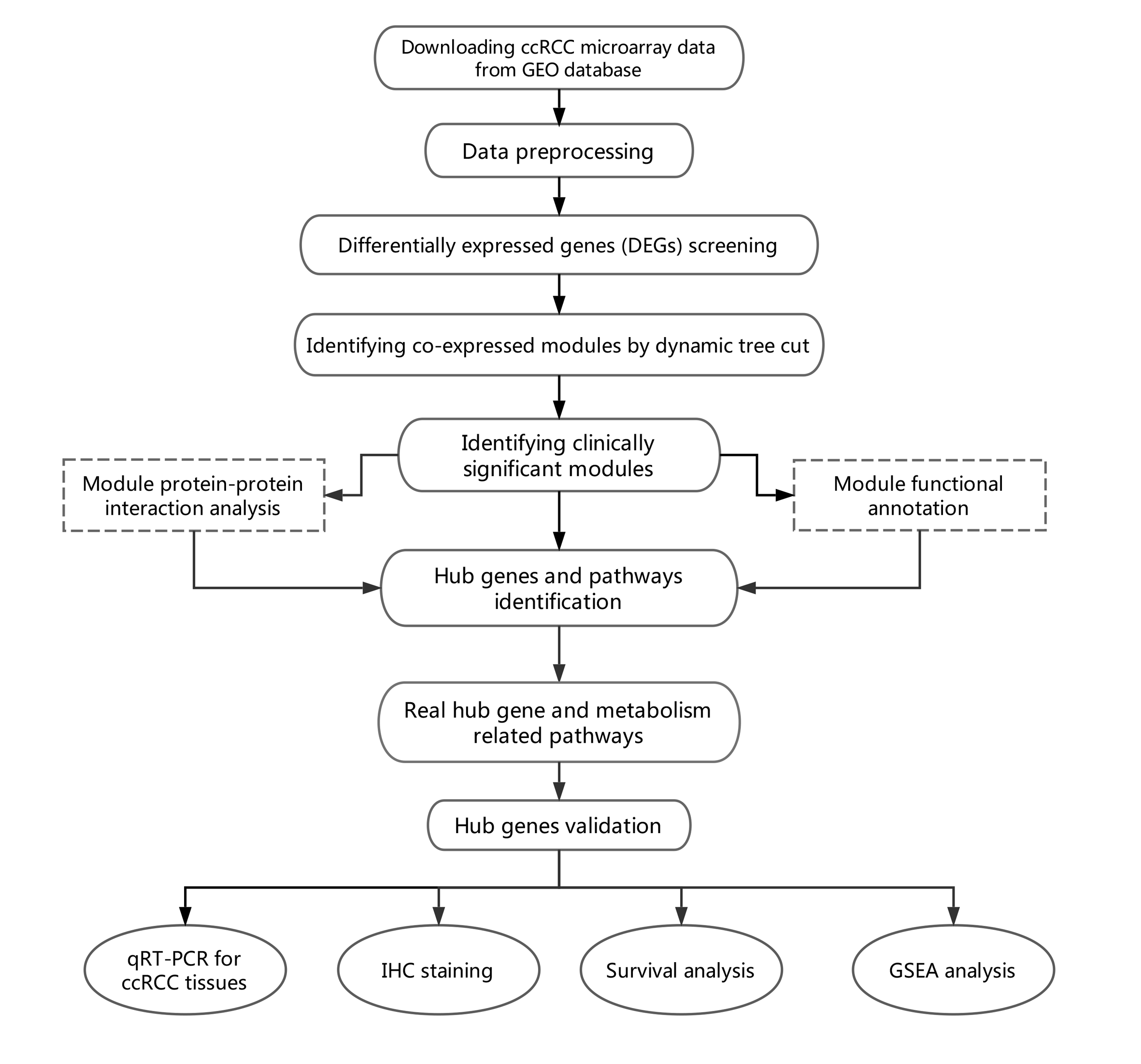

Supplement: Figure S1 — Flow chart of data collection, preparation, processing, analysis, and validation in this study. [file Image_1.TIF]

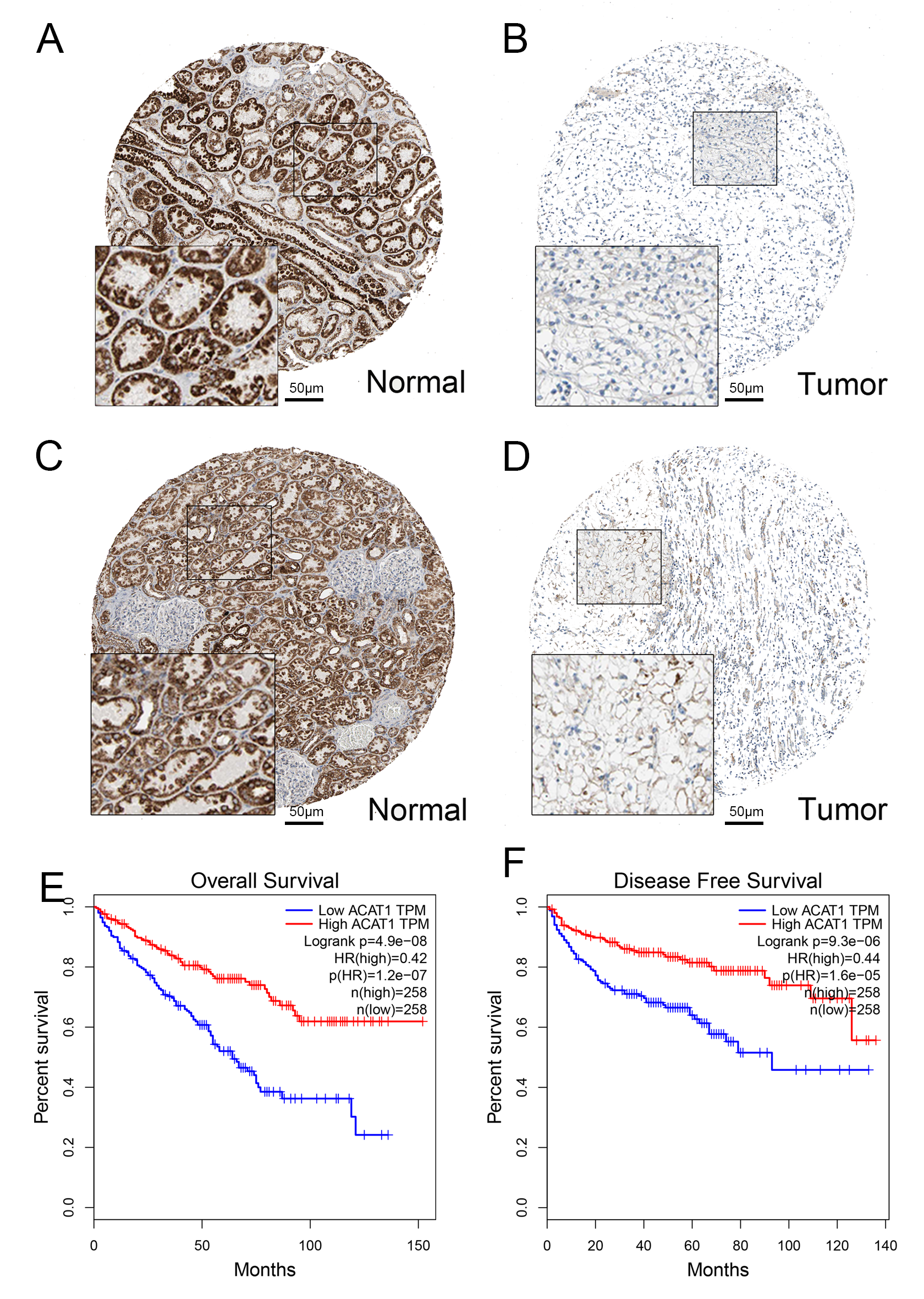

Supplement: Figure S2 — ACAT1 decreased at both the transcriptional and translational levels in ccRCC. (A,C) Immunohistochemistry staining of ACAT1 in normal kidney tissues. (B,D) Immunohistochemistry staining of ACAT1 in ccRCC tissues. (E,F) Kaplan-Meier plot of overall survival time (E) and disease-free survival time (F) for ACAT1 based on TCGA data in the GEPIA database. [file Image_2.TIF]
